# Development and Validation of a Literature Screening Tool: Few-Shot Learning Approach in Systematic Reviews

**DOI:** 10.2196/56863

**Published:** 2024-12-11

**Authors:** Phongphat Wiwatthanasetthakarn, Wanchana Ponthongmak, Panu Looareesuwan, Amarit Tansawet, Pawin Numthavaj, Gareth J McKay, John Attia, Ammarin Thakkinstian

**Affiliations:** 1 Department of Clinical Epidemiology and Biostatistics Faculty of Medicine Ramathibodi Hospital Mahidol University Bangkok Thailand; 2 Department of Research and Medical Innovation Faculty of Medicine Vajira Hospital Navamindradhiraj University Bangkok Thailand; 3 Centre for Public Health Queen’s University Belfast Belfast United Kingdom; 4 Centre for Clinical Epidemiology and Biostatistics School of Medicine and Public Health University of Newcastle New South Wales Australia

**Keywords:** few shots learning, deep learning, natural language processing, S-BERT, systematic review, study selection, sentence-bidirectional encoder representations from transformers

## Abstract

**Background:**

Systematic reviews (SRs) are considered the highest level of evidence, but their rigorous literature screening process can be time-consuming and resource-intensive. This is particularly challenging given the rapid pace of medical advancements, which can quickly make SRs outdated. Few-shot learning (FSL), a machine learning approach that learns effectively from limited data, offers a potential solution to streamline this process. Sentence-bidirectional encoder representations from transformers (S-BERT) are particularly promising for identifying relevant studies with fewer examples.

**Objective:**

This study aimed to develop a model framework using FSL to efficiently screen and select relevant studies for inclusion in SRs, aiming to reduce workload while maintaining high recall rates.

**Methods:**

We developed and validated the FSL model framework using 9 previously published SR projects (2016-2018). The framework used S-BERT with titles and abstracts as input data. Key evaluation metrics, including workload reduction, cosine similarity score, and the number needed to screen at 100% recall, were estimated to determine the optimal number of eligible studies for model training. A prospective evaluation phase involving 4 ongoing SRs was then conducted. Study selection by FSL and a secondary reviewer were compared with the principal reviewer (considered the gold standard) to estimate the false negative rate.

**Results:**

Model development suggested an optimal range of 4-12 eligible studies for FSL training. Using 4-6 eligible studies during model development resulted in similarity thresholds for 100% recall, ranging from 0.432 to 0.636, corresponding to a workload reduction of 51.11% (95% CI 46.36-55.86) to 97.67% (95% CI 96.76-98.58). The prospective evaluation of 4 SRs aimed for a 50% workload reduction, yielding numbers needed to screen 497 to 1035 out of 995 to 2070 studies. The false negative rate ranged from 1.87% to 12.20% for the FSL model and from 5% to 56.48% for the second reviewer compared with the principal reviewer.

**Conclusions:**

Our FSL framework demonstrates the potential for reducing workload in SR screening by over 50%. However, the model did not achieve 100% recall at this threshold, highlighting the potential for omitting eligible studies. Future work should focus on developing a web application to implement the FSL framework, making it accessible to researchers.

## Introduction

The evidence generated from systematic reviews (SRs) and meta-analyses of the published literature is considered to reflect the pinnacle of the evidence hierarchy pyramid [1,2], which in part explains the approximately 20-fold increase in published SRs between 2000 and 2019 [3]. However, conducting an SR requires significant time and human resources, particularly for the screening and selection of potentially eligible studies, data extraction, and bias assessment [4]. Furthermore, some SRs can be outdated by the time of publication, especially in highly progressive medical fields [5].

Multiple automated artificial intelligence (AI) tools have been developed using natural language processing techniques (eg, Abstrackr [6], DistillerSR [7], EPPI-reviewer [8], Rayyan [9], and Covidence [10]) to facilitate the SR processes, particularly study screening. Although the use of automated tools remains controversial [11], the PRISMA (Preferred Reporting Items for Systematic Reviews and Meta-Analyses) 2020 guideline [12] considers them a valuable tool given their potential to significantly reduce screening time or workload [13-18].

These tools [6-10] have largely been developed using supervised machine learning approaches with various document representation techniques and active learning frameworks, which typically require a considerable number of annotated studies as a training set. For example, Abstrackr typically requires 100 studies or more that have been manually screened by a reviewer before making a prediction [13]. During the model training process, iterative annotation of eligible and ineligible studies is required for improved model performance, with the AI model ranking or reordering studies according to their relevance. Subsequently, reviewers can choose between excluding all ineligible AI-predicted studies or simply using the predictions as a guide. The drawback of this approach is that reviewers must annotate studies without previous knowledge of the sufficient number of studies required for model training.

“Few-shot learning” (FSL) is a supervised machine learning approach that can learn from a small number of samples for model training and generalize from limited data [19]. Unlike traditional machine learning, it typically requires large datasets for high accuracy. FSL relies on metric learning during training to measure the similarity between new samples (unseen data) and known samples. Recently, FSL approaches have been successfully applied in many research areas, including computer vision, robotics, and natural language processing [19]. In addition, FSL has been used for concept extraction in health care, such as named entity recognition and text classification [20]. Therefore, the FSL approach is potentially useful for the development of SRs, particularly for study eligibility screening. Only a small number of studies need to be identified for training the FSL framework; theoretically, this approach should make machine learning faster at identifying the most relevant studies for SR compared with traditional approaches.

To the best of our knowledge, the use of FSL has yet to be applied as an automated tool for screening studies for SRs. Therefore, this study aimed to develop a new automated framework using FSL to facilitate the SR screening process with similarly high performance to traditional approaches. The model was trained and evaluated using previously published SRs completed within our institute and was prospectively validated.

## Methods

### Model Development and Validation Phase

#### Data Source

A total of 9 SRs [21-29], hereafter called SR1-9, were used to develop and validate the FSL framework. These SRs covered a range of SR topics including therapy (n=4), prognosis or risk (n=2), genetic association (n=2), and economic evaluation (n=1). All were conducted and published by researchers from the Department of Clinical Epidemiology and Biostatistics, Faculty of Medicine Ramathibodi Hospital, Mahidol University between 2016 and 2018. This diverse set of 9 SRs was chosen to encompass various types of SRs, providing a comprehensive dataset to evaluate the flexibility and robustness of the FSL framework. The total number of identified studies for the 9 SRs ranged between 426 and 7341, of which 9 to 48 studies met the eligibility criteria for individual SRs (Table 1). Titles and abstracts of the studies identified were used as input for model training; the median number of tokens (words) per individual study ranged between 244 and 305. For each SR, the principal and second reviewers selected studies based on individual eligibility criteria. These reviewers included experts in specific areas, such as general physicians, surgeons, pharmacists, and clinical epidemiologists.

**Table 1:** Individual systematic review characteristics for model development and prospective evaluation.

Study	Project name	Study type	Number of studies	Total words (Vocabulary size)	Words per study median (IQR) (range)	Principal reviewer or secondary reviewer background
SR^a^1	Mesh Position for Hernia Prophylaxis After Midline Laparotomy: A Systematic Review and Network Meta-Analysis of Randomized Clinical Trials [21]	Therapeutic studies	3966	1,120,513 (37,777)	285 (206-343) (32-1629)	Surgeon
SR2	The Efficacy of Antibiotic Treatment versus Surgical Treatment of Uncomplicated Acute Appendicitis: Systematic Review and Network Meta-Analysis of Randomized Controlled Trial [22]	Therapeutic studies	1702	411,157 (18,174)	244 (164-307) (37-877)	Epidemiologist or surgeon
SR3	Efficacy and Safety of Urate-Lowering Agents in Asymptomatic Hyperuricemia: Systematic Review and Network Meta-Analysis of Randomized Controlled Trials [23]	Therapeutic studies	7341	2,205,482 (71,645)	297 (214-364) (20-1385)	Internist or pharmacist
SR4	Efficacy and Safety of Antiviral Agents in the Prophylaxis and Pre-Emptive Strategies for Cytomegalovirus Infection on Kidney Transplantation: A Systematic Review and Network Meta-Analysis [24]	Therapeutic studies	3144	874,261 (30,226)	272 (195-337) (33-3509)	Pharmacist or internist
SR5	Association Between Vitamin D and Uric Acid in Adults: A Systematic Review and Meta-Analysis [25]	Prognostic or risk studies	699	191,189 (15,298)	274 (189-340) (20-821)	Physician
SR6	Prognostic Model of Complications in Type 2 Diabetes: Systematic Review and Meta-Analysis [26]	Prognostic or risk studies	426	125,362 (10,843)	292 (232-339) (17-1112)	Epidemiologist
SR7	The Association Between Genetic Polymorphisms in ABCG2 and SLC2A9 and Urate: An Updated Systematic Review and Meta-Analysis [27]	Genetic association studies	1708	444,383 (26,883)	259 (204-312) (29-635)	Pharmacist
SR8	AHSG Gene Polymorphisms, Serum Fetuin-A Levels and Association with Type 2 Diabetes and Cardiovascular Diseases: A Systematic Review and Meta-Analysis [28]	Genetic association studies	1053	318,339 (18,787)	305 (255-346) (70-647)	Physician
SR9	Evaluation of the Cost Utility of Phosphate Binders as a Treatment Option for Hyperphosphatemia in Chronic Kidney Disease Patients: A Systematic Review and Meta-Analysis of the Economic Evaluations [29]	Economic evaluation studies	1653	463,892 (30,602)	244 (176-322) (18-2542)	Pharmacist
Prospective evaluation SR1	Efficacy of EGFR-TKIs Targeted Therapy as Adjuvant Systemic Treatment for Non-Small Cell Lung Cancer: A Systematic Review and Meta-Analysis [30]	Therapeutic studies	1061	386,456 (21,281)	314 (225-442) (48-2553)	Pulmonologist or oncologist
Prospective evaluation SR2	Effect of Repetitive Peripheral Magnetic Stimulation on Upper Extremity Function After Stroke: A systematic review and meta-analysis [31]	Therapeutic studies	1699	561,904 (22,733)	307 (254-362) (19-4422)	Rehabilitation physician or obstetrician
Prospective evaluation SR3	Regular versus As-needed treatments for mild asthma in children, adolescents, and adults: A systematic review and meta-analysis [32]	Therapeutic studies	2136	752,467 (26,540)	324 (271-384) (20-2539)	Oncologist or pulmonologist
Prospective evaluation SR4	The association between cervical sonographic and successful induction of labor: A Systematic review and meta-analysis [33]	Prognostic or risk studies	1646	522,366 (22,494)	301 (243-361) (44-6122)	Obstetrician or rehabilitation physician

^a^SR: systematic review.

#### Data Splitting

In each SR, identified studies were dichotomized as either eligible or ineligible, denoted as S_+_ and S_–_, respectively. Studies were subdivided into training, validation (for model tuning), and test pools according to the following steps (Figure S1 in Multimedia Appendix 1). First, each eligible study 
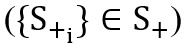
 was randomly assigned to the training 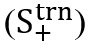, validation 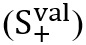, or test 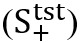 pools with a split ratio of 50%:25%:25%. The eligible studies in the data pools were denoted as 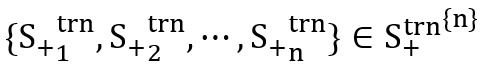 for the training pool, 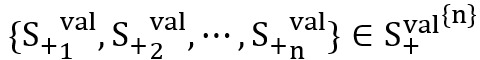 for the validation pool, and 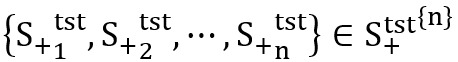 for the test pool, where n is the number of eligible studies in the pool. Second, training and validation pools included the integration of ineligible studies 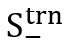 and 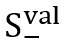, which were randomly selected from the overall S_–_. The number of ineligible studies 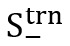 and 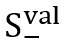 included in the training and validation pools was ten times higher than the eligible studies 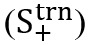 and 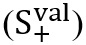, that is, in a ratio of 1:10 representing 1 eligible per 10 ineligible studies. Finally, the remaining ineligible studies were assigned as 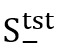 and then combined with 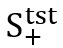 to form the test pool. The ineligible studies in training, validation, and test pools could be denoted as 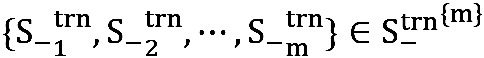, 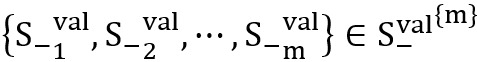, and 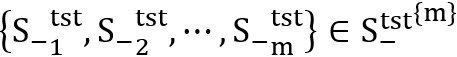, respectively, where m is the number of ineligible studies in each pool.

#### Experimental Scenarios

Multiple scenarios for each SR were tested by varying the number of studies included in 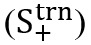 and 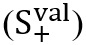 as follows:

- Using all studies in both training and validation pools (scenario 1).
- Reducing S_+_ iteratively by 10% in the training and validation pools (scenario 2, 3, …, i) to a minimum number of 2 in S_+_ (as training data requires a minimum of one positive paired sample in the model framework [refer to Data pairing section]). For 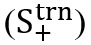 and 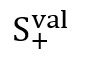 selection in scenario 2 to scenario i, the Euclidean distance between the centroid (ie, the estimated center of all S_+_ in the vector space) and individual S_+_ was used as a criterion for the iterative exclusion of S_+_ from the training and validation pools. The S_+_ farthest from the centroid was initially excluded for each iteration.

Furthermore, the S_–_ number for both pools was reduced to maintain a constant ratio (ie, 1:10) of S_+_ and S_–_. In the test pools, the numbers of 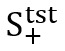 and 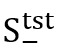 remained the same as scenario 1 for all scenarios to determine the optimal number of eligible studies required for model training and validation.

#### Data Pairing

The model was trained using the Sentence-Bidirectional Encoder Representation for Transformer (S-BERT) [34] which requires paired samples for input data that were generated from each data pool (ie, training, validation, and test pools). For instance, each 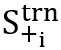 was paired with 
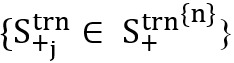
 and 
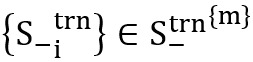
; 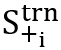 and 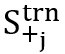 pairs were labeled as 1 (ie, positive pairs), whereas 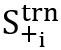 and 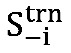 pairs were labeled as 0 (ie, negative pairs). The same pairing process was also performed in the validation set and used for model tuning. All potential combinations of positive paired samples were represented by 
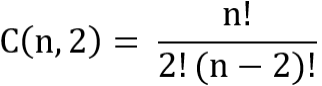
, where 
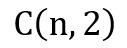
 is a combination of positive paired samples, and the number of eligible studies. For instance, the scenario with 6 eligible studies will have 60 ineligible studies. A training dataset will consist of 4 and 40 eligible and ineligible studies, respectively. These could be paired as 6 positive pairs and 160 negative pairs with a total of 166 paired samples (Figure S2 in Multimedia Appendix 1). A validation dataset includes 2 eligible and 20 ineligible studies with a total of 41 paired samples. The remaining studies were used as test data.

#### Model Architecture and Word Embedding

This study adopted an FSL framework that required several samples for initial model training. We used S-BERT, a Siamese networks architecture model [35] (Figure S3 in Multimedia Appendix 1), for model training with pretrained weighting of “all-mpnet-base-v2,” available on Hugging Face [36]. Each paired sample represented 2 individual studies, study A and B. Raw text data (ie, title and abstract) from each study were fed into S-BERT and transformed into a numerical vector represented by a size of 1 × 384 dimensions. The cosine similarity score was computed from the vector representations of studies A and B, normalized to a scale between 0 and 1 to align contrastive loss in model training. This training approach learns vector representations that bring similar data points closer together and push dissimilar data points further apart. The raw text for each study included was truncated to a maximum length of 384 tokens (words) for S-BERT.

#### Experiment

The FSL model framework was trained separately for each SR project, with training tailored to the specific number of paired samples for each scenario. For instance, in a scenario involving 6 eligible and 60 ineligible studies, the dataset was structured as follows:

- Training data: 4 eligible and 40 ineligible studies were used, resulting in 6 positive pairs and 160 negative pairs (Figure S2 in Multimedia Appendix 1).
- Validation data: 2 eligible and 20 ineligible studies were used, resulting in a total of 41 paired samples.
- Test data: data from the remaining studies were used for testing.

This ensured models were trained and evaluated on datasets specifically tailored to the characteristics of each SR project. The experiments consisted of the following steps:

#### Determination of the Optimal Number of Eligible Studies for Model Training

For each experimental scenario, the number needed to screen (NNS) and %Reduced workload, also known as work, saved oversampling [37], were estimated using the following formulae:



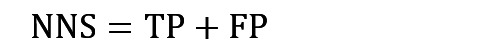





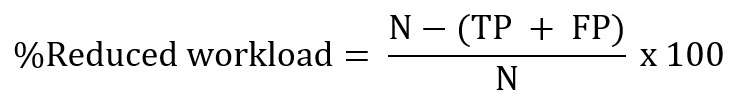



where TP and FP are true and false positive studies (predicted eligible studies), and N is the total number of studies for each SR. The lower the value for the NNS, the higher the %Reduced workload. The %Reduced workload was estimated by fixing the recall rate at 100% (ie, sensitivity), plotted against the number of eligible studies used for training (N positive) for each scenario. The optimal N positive was estimated using the Kneedle algorithm [38], which identifies the knee point of the graph. This method was chosen because it automatically detects the point where the trend in the data significantly changes, providing a more objective and accurate estimation compared with visual inspection or the elbow method.

A CI [39] for the model performance metric (eg, %Reduced workload, precision, recall, *F*_1_-score) was estimated as follows:



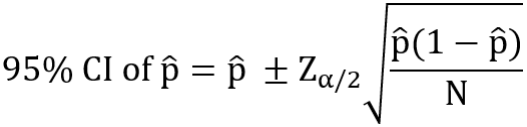



where 
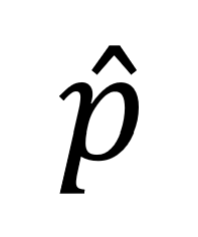
 is the model performance metric, N is the number of studies identified for each SR, Z is a standardized normal distribution, and α is a type I error of .05.

#### Similarity Threshold

Similarity between studies was assessed using a cosine similarity threshold that represented the distance between 2 vector representations for each study within a paired sample. For example, a positive paired sample in a training set consists of 2 eligible studies 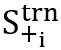 and 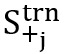. Each study was transformed into a vector representation with a dimension of 1 × 384. The cosine similarity score [40] was calculated using the following equation:



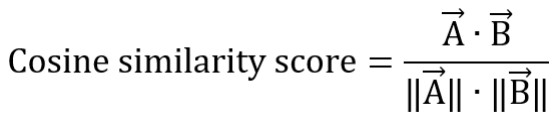



where 
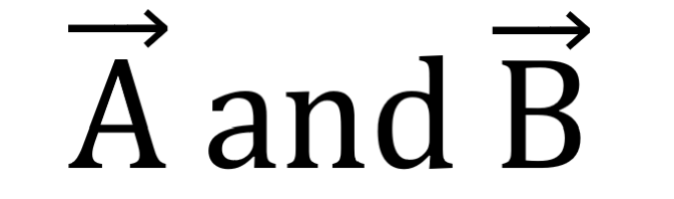
 are vector representations of the first and second studies within a paired sample.

The cosine similarity score ranges from 1 to –1, where 1 represents perfect similarity between both studies and –1 represents complete dissimilarity enabling quantification of the degree of similarity between pairs of studies and the identification of potentially relevant studies based on their vector representations.

Identification of the optimal similarity threshold was based on the %Reduced workload as described in the previous phase, and the feasibility and information available from each of the SR projects that included 4 to 6 eligible studies and a 10 times greater number of ineligible studies. The data from the remaining studies were used as a query set (ie, test pool).

Following the completion of model training, a support set was retrieved from 
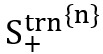
. Each 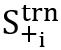 was again paired with all studies from the query set (ie, Q_1_, Q_2_, Q_3_, …, and Q_n_; Figure S4 in Multimedia Appendix 1). The average cosine similarity score for all pairs from the first query study (Q_1_) for each 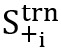 was calculated. For instance, the similarity score of Q_1_ represented an average cosine similarity score of paired samples between Q_1_ and each supporting study set 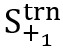, 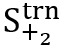, 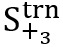, and 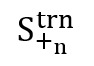. The average cosine similarity score for each query study set (ie, Q_1_, Q_2_, Q_3_, …, Q_n_) was ordered from highest to lowest, and an optimal threshold was selected to achieve 100% recall (ie, the lowest average similarity score for the eligible studies included).

### Prospective Evaluation Phase

Our study prospectively evaluated the FSL framework on four ongoing SR projects (Prospective evaluation SR [PESR] 1-4; Table 1). For each PESR, the principal reviewer initially selected 6 eligible and 60 ineligible studies (4 eligible and 40 ineligible studies for training, and 2 eligible and 20 ineligible studies for validation), leaving the remaining studies in the query set for testing. This initial selection was checked and confirmed by a third senior reviewer and served as the training basis for our FSL framework. The principal reviewer later completed the entire study selection process, providing the “ground truth” for our evaluation.

The FSL framework was applied to rank studies based on average similarity scores. The top-ranked 50% were positive studies representing a potential 50% workload reduction, in contrast to the bottom-ranked 50% which were predicted as negative studies. Positive studies were then allocated to a secondary reviewer for independent screening.

The performance was evaluated with the following 2 steps (Table S1 in Multimedia Appendix 1): (1) comparing the FSL framework model’s output (positive and negative studies) with the principal reviewer’s selections, demonstrating model performance to identify relevant studies and (2) comparing performance between the secondary reviewer and the principal reviewer’s screening based on positive studies by FSL framework. A confusion matrix provided estimates of NNS, recall, precision, and *F*_1_-scores (the harmonic means of precision and recall) for the query set. In addition, false negative rates (FNR), that is, the number of eligible studies misidentified by the FSL framework (not included in the top-ranked 50%, FNR_FSL_) and those not selected by the secondary reviewer (FNR_R2_) were also estimated. As the model was trained on abstract and title data only, the evaluation focused solely on the title and abstract screening.

## Results

### Optimal Number of Eligible Studies (N positive)

Of the 9 SRs, the optimal number of eligible studies (N positive) included in the model training varied between 4 and 12 studies, corresponding to a %Reduced workload of between 80.87% and 99.37% (Figures S5-S8 in Multimedia Appendix 1). A trend for a higher %Reduced workload associated with a higher N positive was observed in SR 1, 3, 4, 5, and 6. In contrast, a higher N positive did not significantly improve %Reduced workload for SRs 2, 7, 8, and 9. However, the optimum N positive ranged between 4 and 12 studies according to the Kneedle algorithm, as represented by the dashed line (Figures S5-S8 in Multimedia Appendix 1). Our findings suggest that the median (range) for the optimum N positive was 9 (4-12) studies, resulting in a median (range) of 95.78% (88.87%-99.37%) %Reduced workload*.* The ideal N positive for feasible model training ranged from 4 to 6, as indicated by the similarity threshold, which is considered acceptable by most reviewers.

### Similarity Threshold

Of the 4 therapeutic SRs (ie, SR1-4), the maximum similarity threshold ranged from 0.439 to 0.617, in line with 100% recall. These threshold values corresponded to a %Reduced workload between 64.81% (95% CI 63.14-66.48) and 96.94% (95% CI 96.12-97.76; Table 2 and Figures S9-S12 in Multimedia Appendix 1). For the prognostic or risk SR5, a similarity threshold of 0.578 was reported equating to a %Reduced workload of 84.16% (95% CI 81.45-86.87). A lower %Reduced workload was observed for prognostic or risk SR6 (51.11%, 95% CI 46.36-55.86 at the similarity threshold of 0.432). For genetic association SRs, the similarity thresholds were 0.546 (SR7) and 0.635 (SR8), corresponding to %Reduced workload of 69.11% (95% CI 66.92-71.30) and 97.67% (95% CI 96.76-98.58), respectively. The similarity threshold and %Reduced workload for the sole economic evaluation SR (SR9) were 0.636 and 95.34% (95% CI 94.32-96.36), respectively. Accordingly, the overall median (range) similarity thresholds and %Reduced workload from our study findings were 0.546 (0.432-0.636) and 84.16% (51.11%-97.67%), respectively.

**Table 2:** Reduced workload and similarity threshold for each systematic review included.

Type of study and project		Optimal similarity score	Reduced workload, % (95% CI)
**Therapeutic study**			
	SR^a^1 [21]	0.439	65.79 (64.31-67.27)
	SR2 [22]	0.617	96.94 (96.12-97.76)
	SR3 [23]	0.544	87.46 (86.70-88.22)
	SR4 [24]	0.539	64.81 (63.14-66.48)
**Prognostic or risk study**			
	SR5 [25]	0.578	84.16 (81.45-86.87)
	SR6 [26]	0.432	51.11 (46.36-55.86)
**Genetic association study**			
	SR7 [27]	0.546	69.11 (66.92-71.30)
	SR8 [28]	0.635	97.67 (96.76-98.58)
**Economic evaluation study**			
	SR9 [29]	0.636	95.34 (94.32-96.36)

^a^SR: systematic review.

### Prospective Evaluation

The prospective evaluation included four PESRs, with PESR1-3 representing therapeutic studies and PESR4 a prognostic or risk study. The total number of studies included ranged from 1061 to 2136, with 49 to 129 of these considered eligible. For model training, 66 studies (6 eligible and 60 ineligible) were initially selected, leaving 995 to 2070 studies for testing, with 43 to 123 eligible studies. Our model reduced the number of overall studies by approximately 50% equating to a workload reduction of 46.84% to 48.46% (ie, NNS divided by total number of studies). The NNS ranged from 497 to 1035, with 40 to 108 eligible studies (Table S2 in Multimedia Appendix 1). With the aspect of the comparison between the FSL framework and principal reviewer, the FNR_FSL_ varied between 1.87% (95% CI 0-4.44) and 12.2% (95% CI 6.42-17.98), producing a recall rate (REC_FSL_) of 87.8% (95% CI 82.02-93.58) to 98.13% (95% CI 95.56-100; Table 3 and Table S2 in Multimedia Appendix 1). The comparison of screening results between the secondary reviewer and the principal reviewer based on positive studies by FSL indicated that PESR1 and PESR4 achieved high recall rates (REC_R2_) of 95% (95% CI 88.25-100) and 88.57% (95% CI 82.48-94.66), respectively, along with FNR_R2_ of 5% (95% CI 0-11.75) and 11.43% (95% CI 5.34-17.52). In contrast, PESR2 and PESR3 indicated high disagreement between both reviewers, with corresponding recall rates (REC_R2_) of 80.88% (95% CI 71.53-90.23) and 43.52% (95% CI 34.17-52.87), with corresponding FNR_R2_ of 19.12% (95% CI 9.77-28.47) and 56.48% (95% CI 47.13-65.83; Table 3 and Tables S2-S6 in Multimedia Appendix 1).

**Table 3:** Performance of prospective systematic review evaluation in the test dataset.

Study type and ID			Few-shot learning versus principal reviewer^a^							Secondary reviewer versus principal reviewer^b^				
			FNR_Few-shot learning_^c^ (95% CI)		REC_Few-shot learning_^d^ (95% CI)		*F*_1__Few-shot learning_^e^ (95% CI)		FNR_secondary reviewer_^c^ (95% CI)			REC_secondary reviewer_^d^ (95% CI)		*F*_1secondary reviewer_^e^ (95% CI)
**Therapeutic study**														
	PESR1^f^ [30]	6.98 (0-14.60)		93.02 (85.40-100)		14.83 (11.71-17.95)		5.00 (0-11.75)			95.00 (88.25-100)		95 (88.41-100)	
	PESR2^f^ [31]	6.85 (1.06-12.64)		93.15 (87.36-98.94)		15.29 (12.83-17.75)		19.12 (9.77-28.47)			80.88 (71.53-90.23)		51.64 (43.85-59.43)	
	PESR3^f^ [32]	12.20 (6.42-17.98)		87.80 (82.02-93.58)		18.63 (16.28-20.98)		56.48 (47.13-65.83)			43.52 (34.17-52.87)		51.65 (43.22-60.08)	
**Prognostic or risk study**														
	PESR4^f^ [33]	1.87 (0-4.44)		98.13 (95.56-100)		23.46 (20.51-26.41)		11.43 (5.34-17.52)			88.57 (82.48-94.66)		88.57 (82.80-94.34)	

^a^Evaluation metrics of few-shot learning framework versus principal reviewer, the model evaluation based on the test data.

^b^Evaluation metrics of secondary reviewer versus principal reviewer, the model evaluation based on number needed to screen.

^c^FNR: false negative rate.

^d^REC: recall.

^e^*F*_1_: *F*_1-_score.

^f^PESR: prospective evaluation systematic review.

## Discussion

### Principal Findings

This study applied an FSL framework to create an automated SR screening tool. The use of 4 to 6 eligible studies for the purpose of model training was sufficient to provide a %Reduced workload between 51.11% and 97.67%, while maintaining 100% recall efficiency. Optimal similarity thresholds varied between 0.432 and 0.636. Paradoxically, increasing the number of eligible studies for model training did not always improve %Reduced workload.

In practice, the principal reviewers undertaking an SR usually perform a preliminary search to identify potentially eligible studies before undertaking a full SR. Considering several eligible studies (4-6 studies) are likely to be identified at this early stage, an FSL approach offers the potential to substantially reduce the subsequent labor-intensive effort required to undertake the SR. The model training within the FSL framework is required once and is generally less time consuming compared with other AI algorithms; for example, existing SR screening tools that use supervised machine learning algorithms with an active learning framework require multiple iterations compared with FSL, which only requires a single training iteration with a small annotated dataset. Subsequently, the trained models are based on those annotated data to predict the remaining studies.

Another aspect of this approach that differs from the existing automated tools involves the use of word embedding (ie, text representation) and classifiers. Several existing tools use text representation techniques that lack context consideration (eg, term frequency), and inverse document frequency with simple classifiers (eg, support vector machine) where performance depends significantly on text representation [6-10]. Instead of using context-free embedding and support vector machine classifiers, this study investigated the feasibility of the use of S-BERT which considers the semantic relationship patterns between a target word and its context before its transformation into a text representation using cosine similarities as a classifier. Thus, FSL frameworks offer improved model performance over currently available SR screening tools.

Among the tools currently available, the performance of Abstrackr has been widely considered with a reported workload reduction between 9.5% and 88.4%, and recall ranging between 79% and 96% [15]. Gates and colleagues also reported a 15%-43% workload reduction with 0%-14% FNR in the subsequent study [18]. Another Abstrackr study [13] reported a 9%-57% workload reduction with associated 0%*-*0.13% FNR. Abstrackr has also been compared with DistillerSR and RobotAnalyst [16], showing a 40%, 49%, and 35% median workload reduction, respectively. With a 100% principal reviewer recall aim (ie, 0% FNR), Tsou et al [17] found a %Reduced workload of 3.99% to 48.41%, and 8.68% to 60.11% for Abstrackr and EPPI-reviewer, respectively. In this context, the FSL framework used in our study improved performance through a %Reduced workload between 51.11% and 97.67%, under the constraints of 100% recall.

We also further prospectively evaluated and validated our FSL framework in 4 additional PESRs. Our FSL framework achieved a 50% workload reduction, with NNS of 497 to 1035 from a total of 995 to 2070 studies after removing 66 studies for training from the initial identified studies. The FNR_FSL_, representing eligible studies missed by the model framework, ranged between 1.87% and 12.2%. This performance was assessed in a prospective evaluation where the principal reviewers initially selected 6 eligible and 60 ineligible studies for training and validation. However, this process differed from the model development phase in several key ways. All studies identified in the development phase were already annotated, whereas only 66 studies were initially annotated when the prospective evaluation experiment began. Guidelines recommend cross-validation between independent reviewer groups for robust SR. Therefore, the potential misidentification of eligible studies by the FSL approach can be mitigated through cross-checking and validation of the principal reviewer’s selection. When comparing the performance of secondary reviewers (ie, evaluating based on the top-ranked 50% of studies by FSL) with the principal reviewer, FNR_R2_ ranged from 5% to 56.48%, which is higher than the corresponding FNR_FSL_, indicating that errors from secondary reviewers contributed significantly to the overall error rate. This emphasizes the importance of training reviewers before and during screening to reduce selection errors. In addition, the involvement of a third senior reviewer is required to resolve these conflicts.

SRs typically require at least 2 independent reviewers for study selection, minimizing potential selection bias as recommended by the PRISMA guidelines [12]. Disagreements between both reviewers are resolved and adjudicated by a third senior reviewer. The aim of our FSL framework is to significantly reduce the workload of the secondary reviewers while still retaining a high recall rate, enabling the secondary reviewer to focus on the studies selected by the FSL framework, rather than having to evaluate all of the studies identified. However, the balance between maximizing workload reduction and minimizing missed eligible studies (ie, FNR_FSL_) must be carefully considered.

When considering the desired workload reduction, researchers should deliberate the complexity of the review topic, including the type of patients, number of treatments, number of genes, number of exposures, and outcomes of interest, which in turn impacts the number of studies identified, which may be subject to personnel resource constraints. For SRs with many identified studies and limited reviewers, a higher workload reduction may be necessary. Conversely, if multiple reviewers are available, the decreased workload reduction required may be less in order to still maintain a reasonable FNR_FSL_. Nevertheless, good practice guidelines recommend cross-validation between independent reviewer groups for robust SR. Therefore, the potential misidentification of eligible studies by the FSL approach can be mitigated through cross-checking and validation of the principal reviewer’s selection.

The trade-off between workload reduction and FNR_FSL_ can vary depending on the type of SR. To assess this variability, our study included a broad range of SRs including therapeutic, risk or prognostic, genetic association, and economic evaluation. While these SRs share a common process, they often involve different patient populations, interventions (eg, treatments, exposures, genes, and costs), and outcomes of interest. For instance, therapeutic studies typically rely on randomized controlled trials with stringent inclusion criteria, while other study types may use cohort data with more flexible inclusion criteria. Model performance is therefore influenced by the complexity of the subject SRs; for example, therapeutic SRs often have clearer eligibility criteria compared with prognostic or risk SRs. Both conceptual approaches resulted in a workload reduction of 64.81%-96.94% for therapeutic SRs and 51.11%-84.16% for prognostic or risk SRs while maintaining 100% recall. Nevertheless, these thresholds are not absolute and depend on the specific complexity of the SR as highlighted.

### Strengths and Limitations

A strength of this study includes the evaluation of the FSL model performance in multiple SR types including therapy, risk or prognosis, genetic association, and economic evaluation. Our FSL framework has the potential to accelerate SR processes during urgent situations, such as public health crises, or in resource-limited settings where efficient resource use is crucial. Furthermore, our framework might be integrated with digital information sources, including eHealth, uHealth, or internet-based medical research, to facilitate study selection for SRs. However, some limitations should be acknowledged. First, the study did not provide model performance in terms of time-saving capabilities. Second, the factors contributing to the worsening recall in the prospective phase were not fully investigated. Both these recognized limitations will be examined in future studies to evaluate the robustness of this approach. Third, the pretrained model requirement was limited to a maximum of 384 words (although, most studies identified across all SRs did not exceed this limit), considering the full texts for each study may not be feasible. Fourth, this study did not evaluate user satisfaction or ease of use. Finally, more SR scenarios are required to prospectively confirm the potential benefits of FSL, with future full-text evaluation compared with the limitations associated with title and abstract screening, although this would likely require the use of high-performance computer processing.

### Directions for Future Work

Our future work will focus on the development of a web application with a simplified usable interface to leverage FSL framework algorithms to streamline literature screening for researchers undertaking SRs to reduce user workload and maintain high-quality study selection using AI-assisted solutions. This will include a more comprehensive prospective evaluation across a wider range of SR types. Furthermore, comparisons of FSL approaches with the integration of large language models to automate study selection and data extraction may reduce the most time-consuming and labor-intensive aspects of SRs and will offer estimates for accuracy in both study selection and data extraction. Automation of these key processes will accelerate the SR workflow and enable researchers to complete more comprehensive and robust reviews with potentially more informative translation into clinical and public health practice.

### Conclusion

In conclusion, the application of FSL approaches for title and abstract screening for undertaking SRs is clearly feasible. The findings from the retrospective evaluation and validation offer promise, although the balance between workload reduction and FNRs for the identification of eligible studies is one that warrants careful consideration.

## Appendix

Multimedia Appendix 1 Additional figures and tables.

Multimedia Appendix 2 Guidelines for developing and reporting machine learning predictive models in biomedical research.

## Abbreviations

AI: artificial intelligence
FNR: false negative rate
FSL: few-shot learning
NNS: number needed to screen
PESR: prospective evaluation systematic review
PRISMA: Preferred Reporting Items for Systematic Reviews and Meta-Analyses
S-BERT: sentence-bidirectional encoder representations from transformers
SR: systematic review
