# Supplementary material for: Development and Validation of a Literature Screening Tool: Few-Shot Learning Approach in Systematic Reviews
Source: J Med Internet Res. 2024 Dec 11;26:e56863. doi: 10.2196/56863 (PMC11669879; doi:10.2196/56863)
Supplement: Multimedia Appendix 1 [file jmir_v26i1e56863_app1.docx]

**Multimedia Appendix 1**

**Table of Figures**

[Supplementary Figure 1. Data splitting method for the designation of training, validation, and test pools. 3](#_Toc178610373)

[Supplementary Figure 2. Example of generating paired training data. 4](#_Toc178610374)

[Supplementary Figure 3. Model architecture of the study (S-BERT). 5](#_Toc178610375)

[Supplementary Figure 4. Sample pairing method for estimation of the average cosine similarity threshold 6](#_Toc178610376)

[Supplementary Figure 5. Evaluation of the number of eligible studies used for training phase and the reduced workload for each therapeutic systematic review project. 7](#_Toc178610377)

[Supplementary Figure 6. Evaluation of the number of eligible studies used for training phase and the reduced workload for each prognosis/risk systematic review project 8](#_Toc178610378)

[Supplementary Figure 7. Evaluation of the number of eligible studies used for training phase and the reduced workload for each genetic association systematic review project. 9](#_Toc178610379)

[Supplementary Figure 8. Evaluation of the number of eligible studies used for training phase and the reduced workload for each economic evaluation systematic review project. 10](#_Toc178610380)

[Supplementary Figure 9. Optimal cosine similarity thresholds at 100% recall for the therapeutic systematic reviews. 11](#_Toc178610381)

[Supplementary Figure 10. Optimal cosine similarity thresholds at 100% recall for the prognostic/risk systematic reviews. 12](#_Toc178610382)

[Supplementary Figure 11. Optimal cosine similarity thresholds at 100% recall for the genetic association systematic reviews. 13](#_Toc178610383)

[Supplementary Figure 12. Optimal cosine similarity thresholds at 100% recall for the economic evaluation systematic reviews. 14](#_Toc178610384)

**Table of Tables**

[Supplementary Table 1. A confusion matrix comparing study selections between the FSL framework model, the secondary reviewer, and the principal reviewer using the test data in the prospective evaluation of a systematic review 15](#_Toc178610385)

[Supplementary Table 6. Performance of prospective systematic review evaluation in the test data set 16](#_Toc178610386)

[Supplementary Table 2. A 2x2 confusion matrix comparing study selections of PESR1 between the secondary reviewer, the FSL framework model, and the principal reviewer using the test data 17](#_Toc178610387)

[Supplementary Table 3. A 2x2 confusion matrix comparing study selections of PESR2 between the secondary reviewer, the FSL framework model, and the principal reviewer using the test data 18](#_Toc178610388)

[Supplementary Table 4. A 2x2 confusion matrix comparing study selections of PESR3 between the secondary reviewer, the FSL framework model, and the principal reviewer using the test data 19](#_Toc178610389)

[Supplementary Table 5. A 2x2 confusion matrix comparing study selections of PESR4 between the secondary reviewer, the FSL framework model, and the principal reviewer using the test data 20](#_Toc178610390)


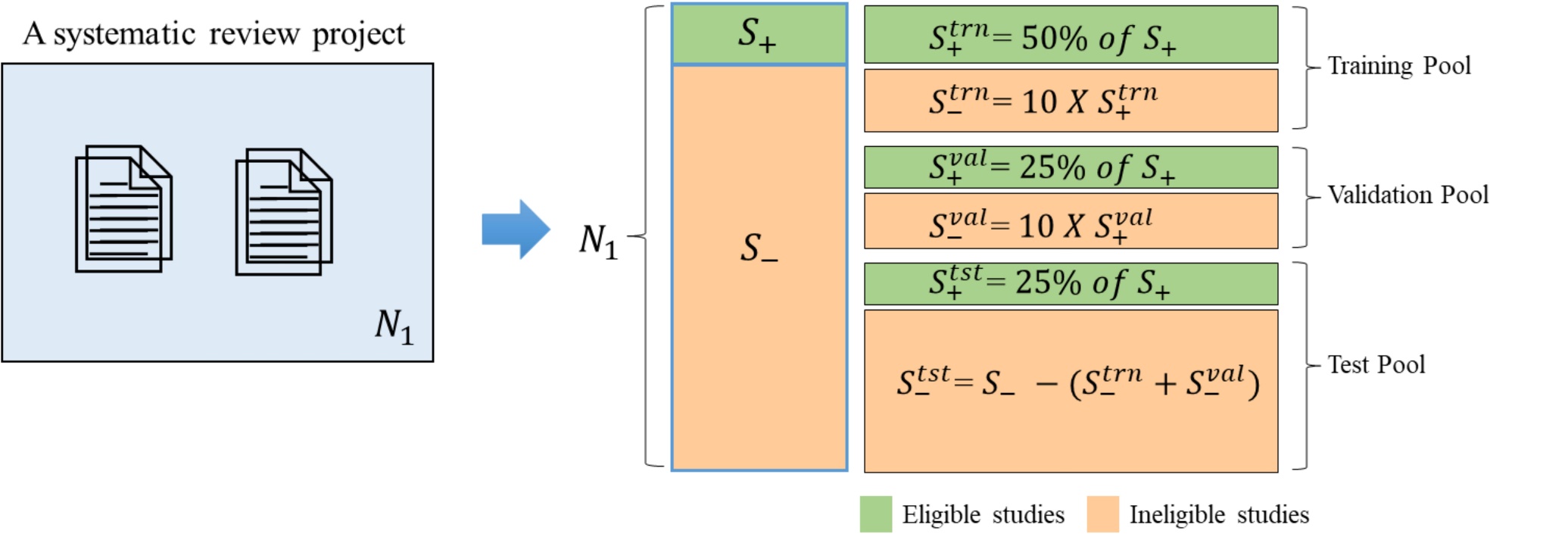


Supplementary Figure 1. Data splitting method for the designation of training, validation, and test pools.


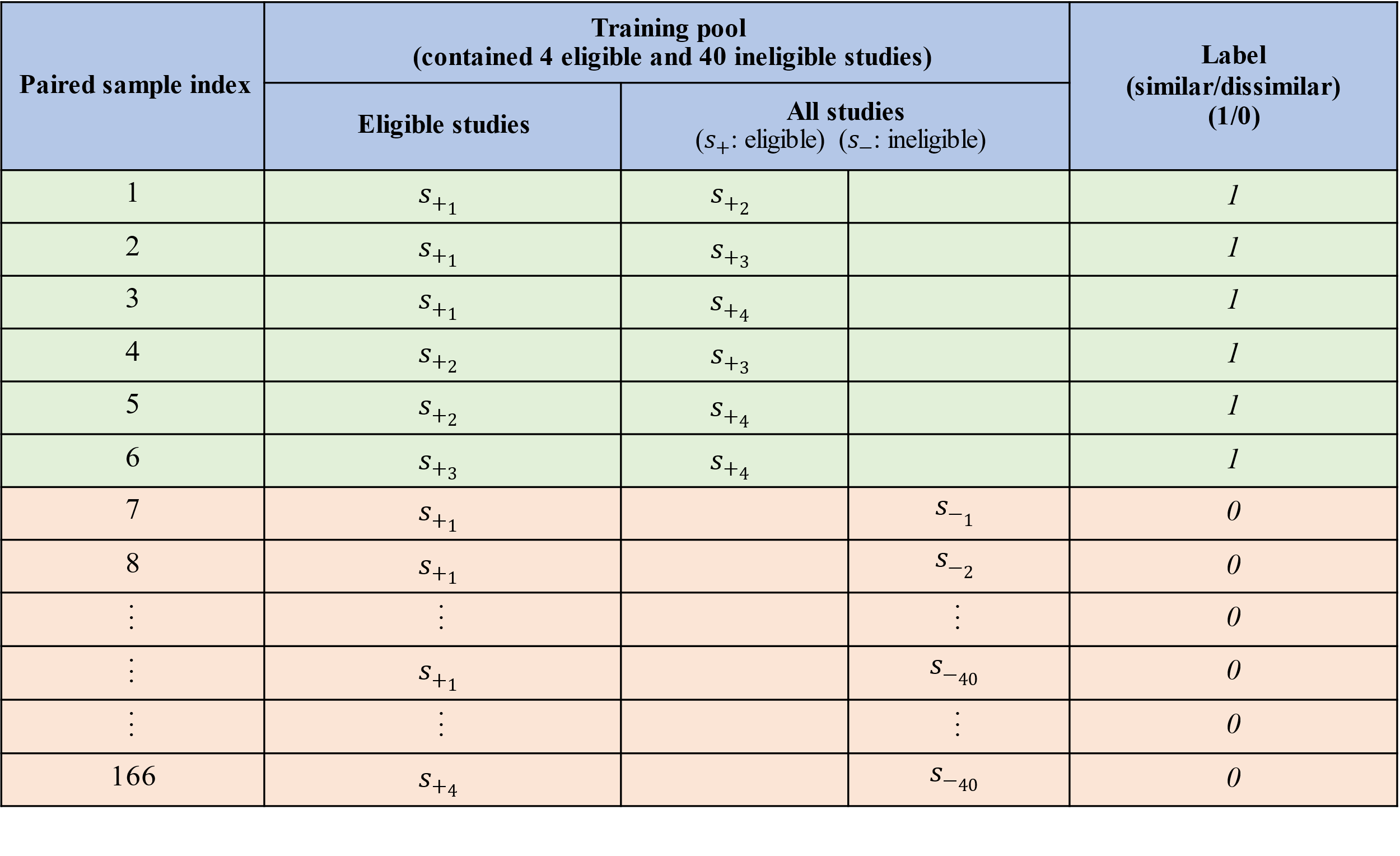


Supplementary Figure 2. Example of generating paired training data.


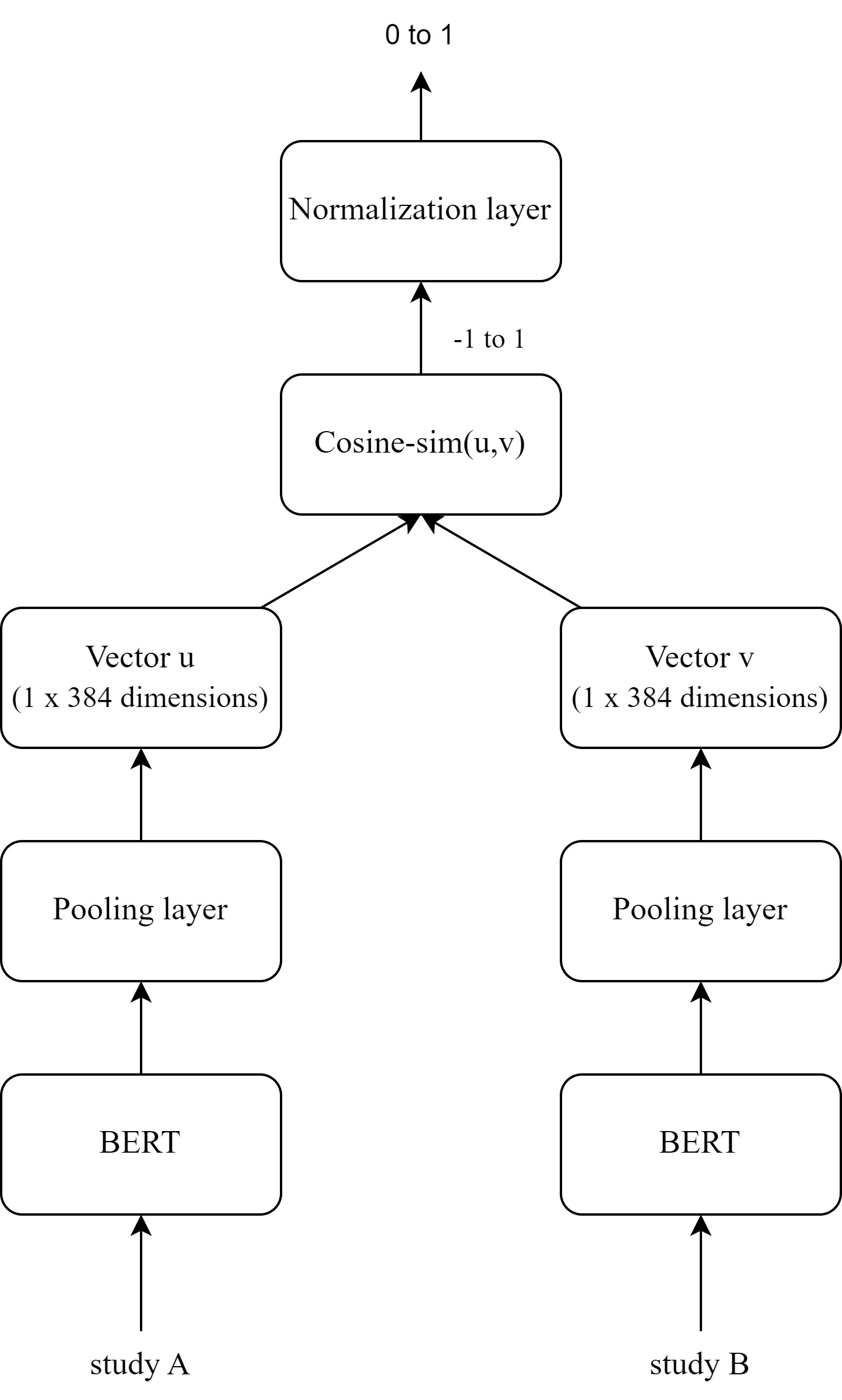


Supplementary Figure 3. Model architecture of the study (S-BERT).

*
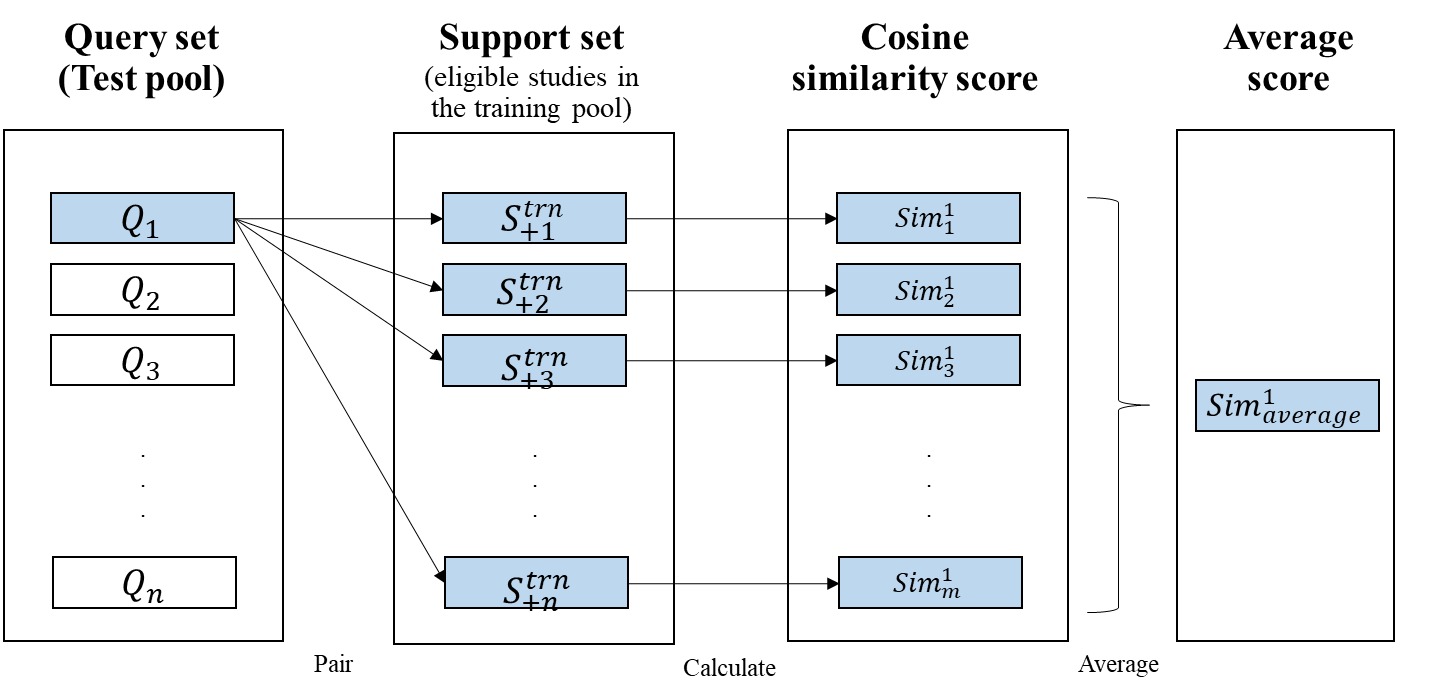
*

Supplementary Figure 4. Sample pairing method for estimation of the average cosine similarity threshold

**
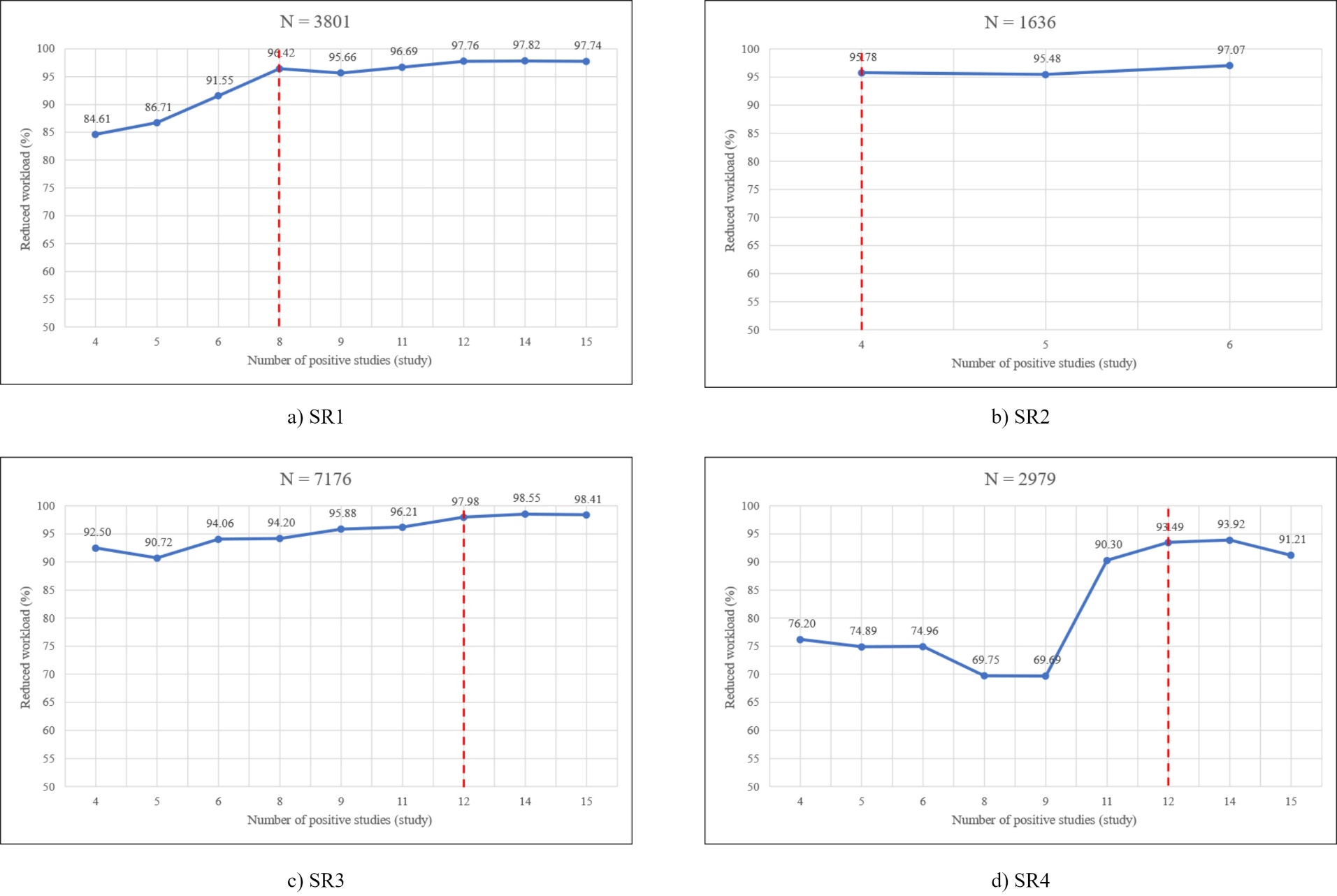
**

Supplementary Figure 5. Evaluation of the number of eligible studies used for training phase and the reduced workload for each therapeutic systematic review project.

*
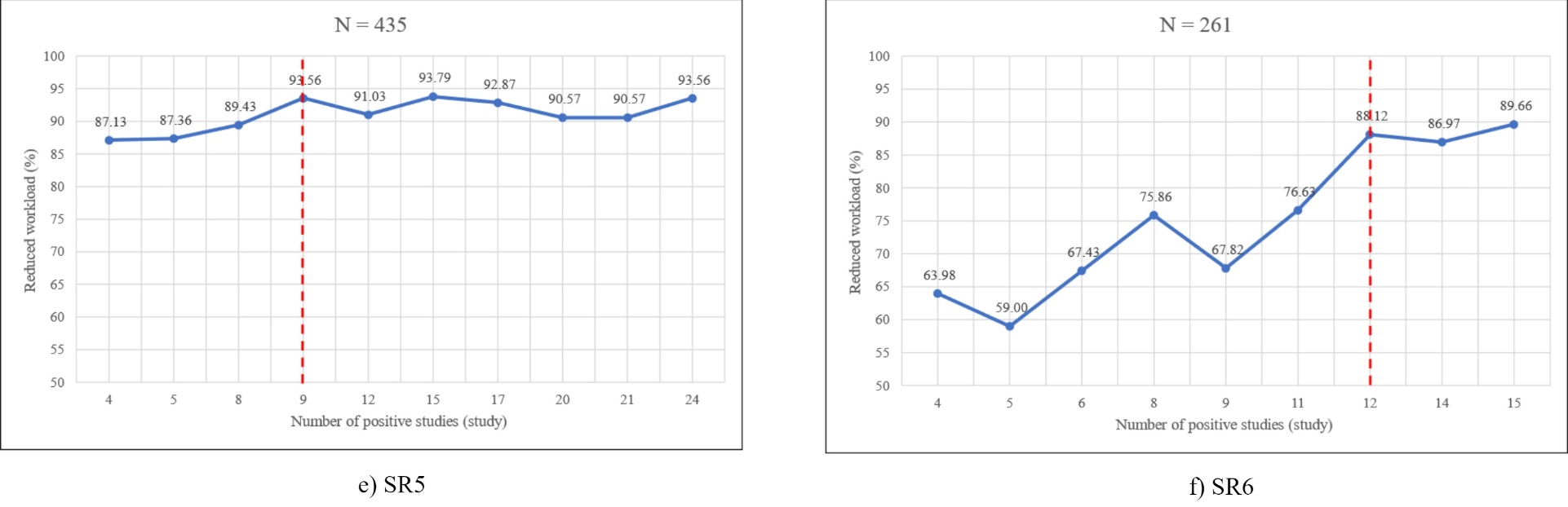
*

Supplementary Figure 6. Evaluation of the number of eligible studies used for training phase and the reduced workload for each prognosis/risk systematic review project

*
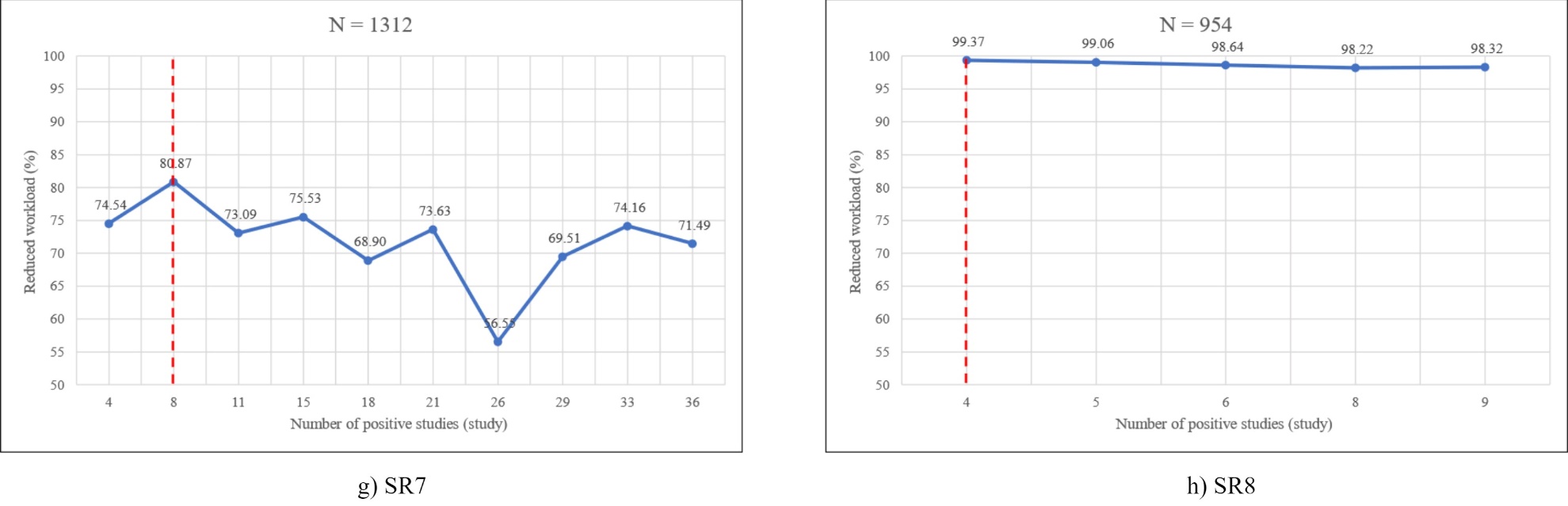
*

Supplementary Figure 7. Evaluation of the number of eligible studies used for training phase and the reduced workload for each genetic association systematic review project.


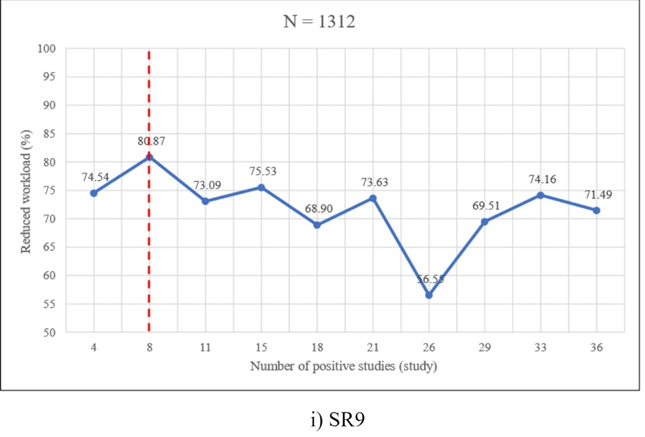


Supplementary Figure 8. Evaluation of the number of eligible studies used for training phase and the reduced workload for each economic evaluation systematic review project.

*
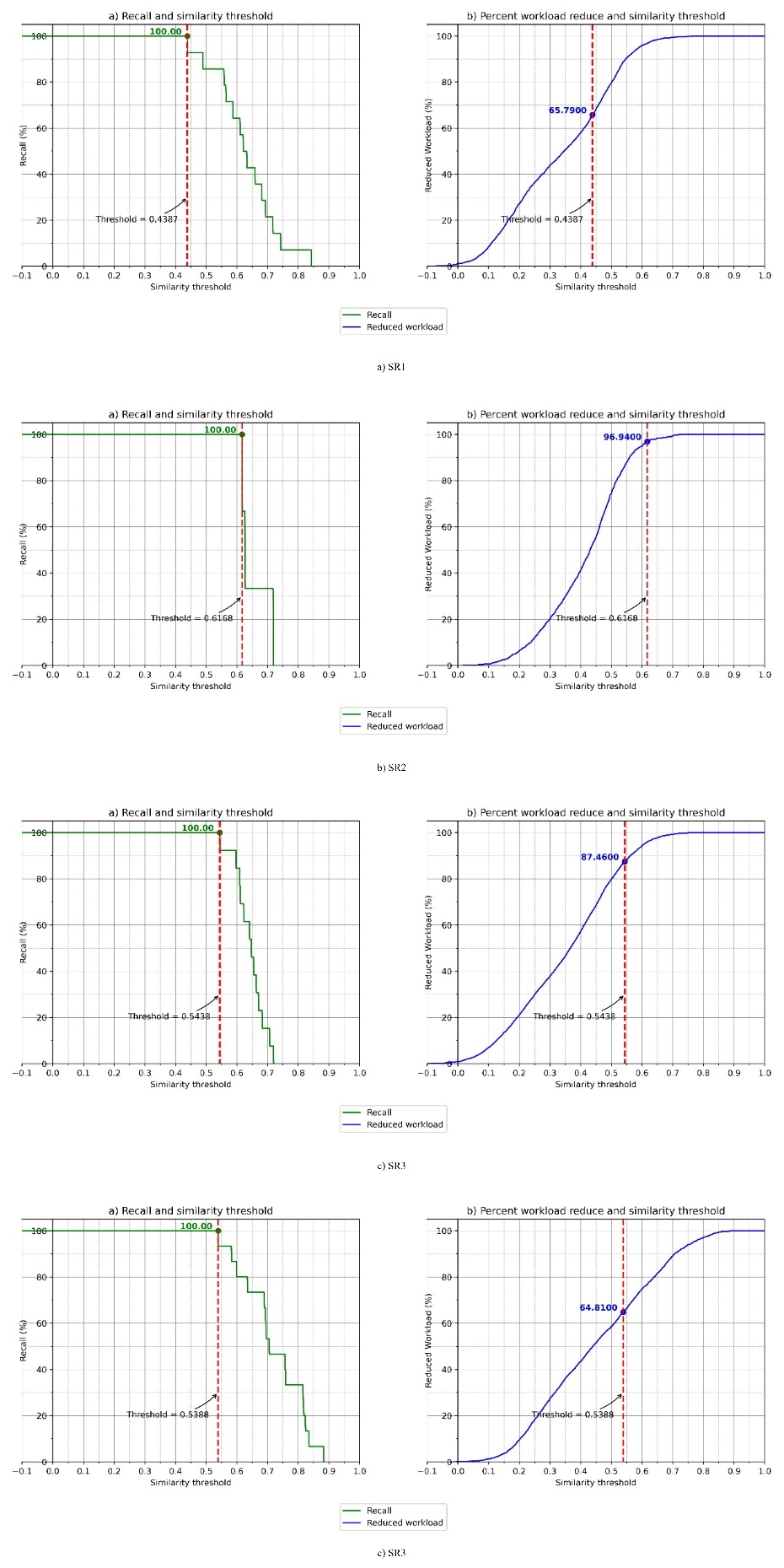
*

Supplementary Figure 9. Optimal cosine similarity thresholds at 100% recall for the therapeutic systematic reviews.

*
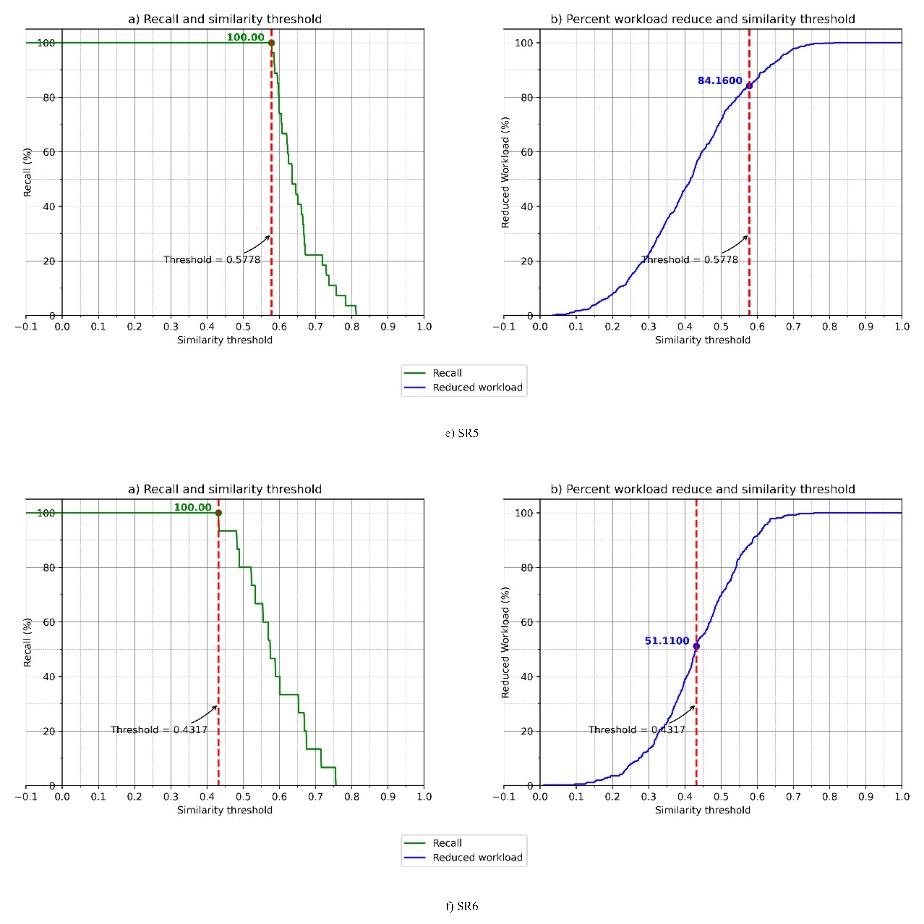
*

Supplementary Figure 10. Optimal cosine similarity thresholds at 100% recall for the prognostic/risk systematic reviews.

*
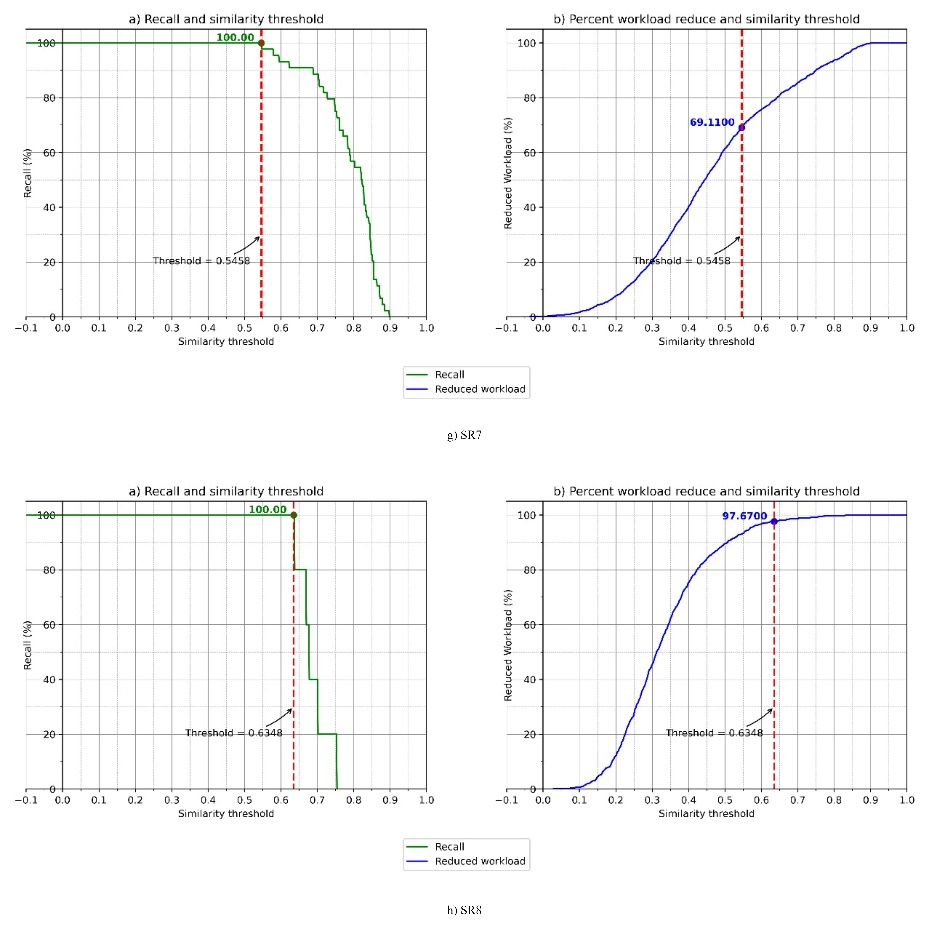
*

Supplementary Figure 11. Optimal cosine similarity thresholds at 100% recall for the genetic association systematic reviews.*
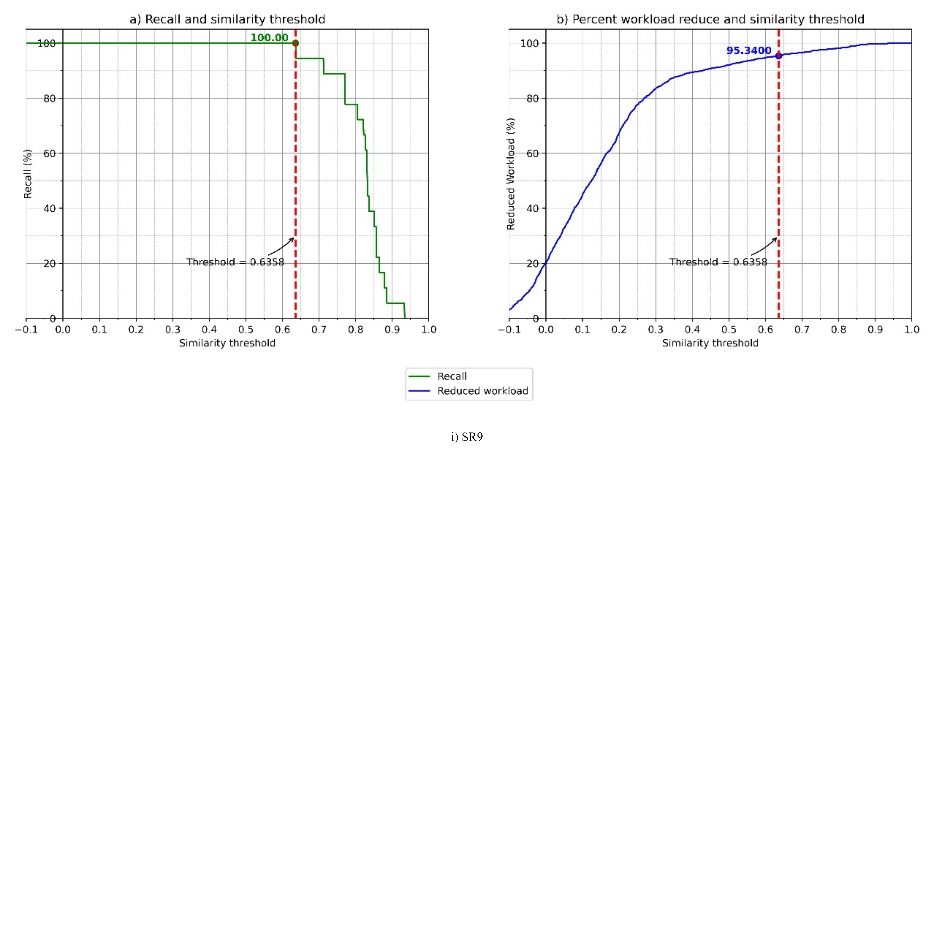
*

Supplementary Figure 12. Optimal cosine similarity thresholds at 100% recall for the economic evaluation systematic reviews.

Supplementary Table 1. A confusion matrix comparing study selections between the FSL framework model, the secondary reviewer, and the principal reviewer using the test data in the prospective evaluation of a systematic review

| FSL framework | Secondary reviewer (R2) | Principal reviewer (R1) | | Total |
| --- | --- | --- | --- | --- |
|  |  | Eligible studies | Ineligible studies |  |
| FSL^+^  (Top-ranked 50%) | Positive | TP_2_ | FP_2_ | $N^{+}$ |
|  | Negative | FN_2_ | TN_2_ |  |
| FSL^-^  (Bottom-ranked 50%) | No screening | FN_1_ | TN_1_ | $N^{-}$ |
| Total |  | TP_2_+FN_1_+FN_2_ | ${FP}_{2}$+TN_1_+TN_2_ | $N^{*}$ |
| ${Recall}_{FSL}$ | $\frac{{TP}_{2}+{FN}_{2}}{{TP}_{2}+{FN}_{2}+{FN}_{1}}$ | | |  |
| ${FNR}_{FSL}$, mis-identified studies by FSL | ${1-Recall}_{FSL}$ | | |  |
| ${Precision}_{FSL}$ | $\frac{{TP}_{2}+{FN}_{2}}{N^{+}}$ | | |  |
| ${F1}_{FSL}$ | $2\times\frac{{Recall}_{FSL}\times{Precision}_{FSL}}{{Recall}_{FSL}+{Precision}_{FSL}}$ | | |  |
| ${Recall}_{R2}$ | $\frac{{TP}_{2}}{{TP}_{2}+{FN}_{2}}$ | | |  |
| ${FNR}_{R2}$, mis-identified studies by R_2_ | ${1-Recall}_{R2}$ | | |  |
| ${Precision}_{R2}$ | $\frac{{TP}_{2}}{{TP}_{2}+{FP}_{2}}$ | | |  |
| ${F1}_{R2}$ | $2\times\frac{{Recall}_{R2}{\times Precision}_{R2}}{{Recall}_{R2}+{Precision}_{R2}}$ | | |  |

N^*^: a total number of studies – 66 studies for model training; TP_2_: the study is eligible by the principal reviewer and appears in the top-ranked 50% of studies (FSL+) and eligible by the second reviewer; FP_2_: the study is ineligible by the principal reviewer and appears in the top-ranked 50% of studies (FSL+) and eligible by the second reviewer; FN_2_: the study is eligible by the principal reviewer and appears in the top-ranked 50% of studies (FSL+) but ineligible by the second reviewer; TN_2_: the study is ineligible by the principal reviewer and appears in the top-ranked 50% of studies (FSL+) but ineligible by the second reviewer; FN_1_: the study is eligible by the principal reviewer and does not appear in the top-ranked 50% of studies (FSL-); TN_1_: the study is ineligible by the principal reviewer and does not appear in the top-ranked 50% of studies (FSL-), FNR: false negative rate.

Supplementary Table 2. Performance of prospective systematic review evaluation in the test data set

| **Type of study** | **Project** | **All data**  **identified/**  **eligible studies**  **(N)** | **Test data^*^**  **identified/**  **eligible studies**  **(N)** | **NNS (%RW)** | **N eligible studies at %RW** | **N mis-identified eligible studies** | **FSL framework vs. PI^**^** | | | | **2^nd^ reviewer (R2) vs. PI^***^** | | | |
| --- | --- | --- | --- | --- | --- | --- | --- | --- | --- | --- | --- | --- | --- | --- |
|  |  |  |  |  |  |  | **%FNR_FSL_**  **(95%CI)** | **%REC_FSL_**  **(95%CI)** | **%PR_FSL_**  **(95%CI)** | **%F1_FSL_**  **(95%CI)** | **%FNR_R2_**  **(95%CI)** | **%REC_R2_**  **(95%CI)** | **%PR_R2_**  **(95%CI)** | **%F1_R2_**  **(95%CI)** |
|  |  |  |  |  |  |  |  |  |  |  |  |  |  |  |
| **Therapeutic study** |  |  |  |  |  |  |  |  |  |  |  |  |  |  |
|  | PESR1 | 1,061/49 | 995/43 | 497 (46.84) | 40 | 3 | 6.98 (0.00-14.60) | 93.02 (85.40-100.00) | 8.06 (5.66-10.46) | 14.83 (11.71-17.95) | 5.00 (0.00-11.75) | 95.00 (88.25-100.00) | 95.00 (88.25-100.00) | 95.00 (88.41-100.00) |
|  | PESR2 | 1,699/79 | 1,633/73 | 816 (48.03) | 68 | 5 | 6.85 (1.06-12.64) | 93.15 (87.36-98.94) | 8.33 (6.43-10.23) | 15.29 (12.83-17.75) | 19.12 (9.77-28.47) | 80.88 (71.53-90.23) | 37.93 (30.03-45.83) | 51.64 (43.85-59.43) |
|  | PESR3 | 2,136/129 | 2,070/123 | 1,035 (48.46) | 108 | 15 | 12.20 (6.42-17.98) | 87.80 (82.02-93.58) | 10.42 (8.56-12.28) | 18.63 (16.28-20.98) | 56.48 (47.13-65.83) | 43.52 (34.17-52.87) | 63.51 (52.54-74.48) | 51.65 (43.22-60.08) |
| **Prognostic/ Risk study** |  |  |  |  |  |  |  |  |  |  |  |  |  |  |
|  | PESR4 | 1,646/113 | 1,580/107 | 790 (48.00) | 105 | 2 | 1.87 (0.00-4.44) | 98.13 (95.56-100.00) | 13.32 (10.95-15.69) | 23.46 (20.51-26.41) | 11.43 (5.34-17.52) | 88.57 (82.48-94.66) | 88.57 (82.48-94.66) | 88.57 (82.8-94.34) |

CI: confidence interval, FNR: false negative rate, FSL: few-shot learning, N: number, NNS: number needed to screen, PESR: prospective evaluation systematic review, PI: Principal reviewer, PR: precision, REC: recall, RW: reduced workload, R2: secondary reviewer.

^*:^ The all data were removed 66 identified studies (6 eligible and 60 ineligible studies) for model training, the rest were test data.

^**:^ FSL framework vs. PI, the model evaluation based on the test data.

^***:^ R2 vs. PI, the model evaluation based on NNS

Supplementary Table 3. A 2x2 confusion matrix comparing study selections of PESR1 between the secondary reviewer, the FSL framework model, and the principal reviewer using the test data

| FSL framework | Secondary reviewer (R2) | Principal reviewer (R1) | | Total |
| --- | --- | --- | --- | --- |
|  |  | Eligible studies | Ineligible studies |  |
| FSL+  (top-ranked 50%) | Positive | 38 | 2 | 40 |
|  | Negative | 2 | 454 | 456 |
| FSL-  (bottom-ranked 50%) | No screening | 3 | 496 | 499 |
| Total |  | 43 | 952 | 995 |
| ${Recall}_{FSL}$ | $\frac{38+2}{38+2+3}\times100=93.02$ | | |  |
| ${FNR}_{FSL}$ | $100-93.02=6.98$ | | |  |
| ${Precision}_{FSL}$ | $\frac{38+2}{40+456}\times100=8.06$ | | |  |
| ${F1}_{FSL}$ | $2\times\frac{93.02\times8.06}{93.02+8.06}=14.83$ | | |  |
| ${Recall}_{R2}$ | $\frac{38}{38+2}\times100=95.00$ | | |  |
| ${FNR}_{R2}$ | $100-95.00=5.00$ | | |  |
| ${Precision}_{R2}$ | $\frac{38}{38+2}\times100=95.00$ | | |  |
| ${F1}_{R2}$ | $2\times\frac{95.00\times95.00}{95.00+95.00}=95.00$ | | |  |

Supplementary Table 4. A 2x2 confusion matrix comparing study selections of PESR2 between the secondary reviewer, the FSL framework model, and the principal reviewer using the test data

| FSL framework | Secondary reviewer (R2) | Principal reviewer (R1) | | Total |
| --- | --- | --- | --- | --- |
|  |  | Eligible studies | Ineligible studies |  |
| FSL+  (top-ranked 50%) | Positive | 55 | 90 | 145 |
|  | Negative | 13 | 658 | 671 |
| FSL-  (bottom-ranked 50%) | No screening | 5 | 812 | 817 |
| Total |  | 73 | 1,560 | 1,633 |
| ${Recall}_{FSL}$ | $\frac{55+13}{55+13+5}\times100=93.15$ | | |  |
| ${FNR}_{FSL}$ | $100-93.15=6.85$ | | |  |
| ${Precision}_{FSL}$ | $\frac{55+13}{145+671}\times100=8.33$ | | |  |
| ${F1}_{FSL}$ | $2\times\frac{93.15\times8.33}{93.15+8.33}=15.29$ | | |  |
| ${Recall}_{R2}$ | $\frac{55}{55+13}\times100=80.88$ | | |  |
| ${FNR}_{R2}$ | $100-80.88=19.12$ | | |  |
| ${Precision}_{R2}$ | $\frac{55}{145}\times100=37.93$ | | |  |
| ${F1}_{R2}$ | $2\times\frac{80.88\times37.93}{80.88+37.93}=51.64$ | | |  |

Supplementary Table 5. A 2x2 confusion matrix comparing study selections of PESR3 between the secondary reviewer, the FSL framework model, and the principal reviewer using the test data

| FSL framework | Secondary reviewer (R2) | Principal reviewer (R1) | | Total |
| --- | --- | --- | --- | --- |
|  |  | Eligible studies | Ineligible studies |  |
| FSL+  (top-ranked 50%) | Positive | 47 | 27 | 74 |
|  | Negative | 61 | 901 | 962 |
| FSL-  (bottom-ranked 50%) | No screening | 15 | 1,019 | 1,034 |
| Total |  | 123 | 1,947 | 2,070 |
| ${Recall}_{FSL}$ | $\frac{47+61}{47+61+15}\times100=87.80$ | | |  |
| ${FNR}_{FSL}$ | $100-87.80=12.20$ | | |  |
| ${Precision}_{FSL}$ | $\frac{47+61}{74+962}\times100=10.42$ | | |  |
| ${F1}_{FSL}$ | $2\times\frac{87.80\times10.42}{87.80+10.42}=18.63$ | | |  |
| ${Recall}_{R2}$ | $\frac{47}{47+61}\times100=43.52$ | | |  |
| ${FNR}_{R2}$ | $100-43.52=56.48$ | | |  |
| ${Precision}_{R2}$ | $\frac{47}{47+27}\times100=63.51$ | | |  |
| ${F1}_{R2}$ | $2\times\frac{43.52\times63.51}{43.52+63.51}=51.65$ | | |  |

Supplementary Table 6. A 2x2 confusion matrix comparing study selections of PESR4 between the secondary reviewer, the FSL framework model, and the principal reviewer using the test data

| FSL framework | Secondary reviewer (R2) | Principal reviewer (R1) | | Total |
| --- | --- | --- | --- | --- |
|  |  | Eligible studies | Ineligible studies |  |
| FSL+  (top-ranked 50%) | Positive | 93 | 12 | 105 |
|  | Negative | 12 | 671 | 683 |
| FSL-  (bottom-ranked 50%) | No screening | 2 | 790 | 792 |
| Total |  | 107 | 1,473 | 1,580 |
| ${Recall}_{FSL}$ | $\frac{93+12}{93+12+2}\times100=98.13$ | | |  |
| ${FNR}_{FSL}$ | $100-98.13=1.87$ | | |  |
| ${Precision}_{FSL}$ | $\frac{93+12}{105+683}\times100=13.32$ | | |  |
| ${F1}_{FSL}$ | $2\times\frac{98.13\times13.32}{98.13+13.32}=23.46$ | | |  |
| ${Recall}_{R2}$ | $\frac{93}{93+12}\times100=88.57$ | | |  |
| ${FNR}_{R2}$ | $100-88.57=11.43$ | | |  |
| ${Precision}_{R2}$ | $\frac{93}{93+12}\times100=88.57$ | | |  |
| ${F1}_{R2}$ | $2\times\frac{88.57\times88.57}{88.57+88.57}=88.57$ | | |  |
